# Quantifying the healthcare costs of treating severely bleeding major trauma patients: a national study for England

**DOI:** 10.1186/s13054-015-0987-5

**Published:** 2015-07-06

**Authors:** Helen E. Campbell, Elizabeth A. Stokes, Danielle N. Bargo, Nicola Curry, Fiona E. Lecky, Antoinette Edwards, Maralyn Woodford, Frances Seeney, Simon Eaglestone, Karim Brohi, Alastair M. Gray, Simon J. Stanworth

**Affiliations:** Health Economics Research Centre, Nuffield Department of Population Health, University of Oxford, Old Road Campus, Headington, Oxford, OX3 7LF UK; Eli Lilly and Company Limited, Lilly House, Priestley Road, Basingstoke, Hampshire RG24 9NL UK; Oxford Haemophilia and Thrombosis Centre, Oxford University Hospitals NHS Trust, Churchill Hospital, Oxford, OX3 7LE UK; Trauma Audit and Research Network, 3rd Floor Mayo Building, Salford Royal NHS Foundation Trust, Salford, M6 8HD UK; NHS Blood and Transplant Clinical Trials Unit, Fox Den Road, Stoke Gifford, Bristol, BS34 8RR UK; Blizard Institute, Barts and The London School of Medicine and Dentistry, The Blizard Building, 4 Newark Street, London, E1 2AT UK; NHS Blood and Transplant and Oxford University Hospitals NHS Trust, John Radcliffe Hospital, Headley Way, Headington, Oxford, OX3 9BQ UK

## Abstract

**Introduction:**

Severely bleeding trauma patients are a small proportion of the major trauma population but account for 40 % of all trauma deaths. Healthcare resource use and costs are likely to be substantial but have not been fully quantified. Knowledge of costs is essential for developing targeted cost reduction strategies, informing health policy, and ensuring the cost-effectiveness of interventions.

**Methods:**

In collaboration with the Trauma Audit Research Network (TARN) detailed patient-level data on in-hospital resource use, extended care at hospital discharge, and readmissions up to 12 months post-injury were collected on 441 consecutive adult major trauma patients with severe bleeding presenting at 22 hospitals (21 in England and one in Wales). Resource use data were costed using national unit costs and mean costs estimated for the cohort and for clinically relevant subgroups. Using nationally available data on trauma presentations in England, patient-level cost estimates were up-scaled to a national level.

**Results:**

The mean (95 % confidence interval) total cost of initial hospital inpatient care was £19,770 (£18,177 to £21,364) per patient, of which 62 % was attributable to ventilation, intensive care, and ward stays, 16 % to surgery, and 12 % to blood component transfusion. Nursing home and rehabilitation unit care and re-admissions to hospital increased the cost to £20,591 (£18,924 to £22,257). Costs were significantly higher for more severely injured trauma patients (Injury Severity Score ≥15) and those with blunt injuries. Cost estimates for England were £148,300,000, with over a third of this cost attributable to patients aged 65 years and over.

**Conclusions:**

Severely bleeding major trauma patients are a high cost subgroup of all major trauma patients, and the cost burden is projected to rise further as a consequence of an aging population and as evidence continues to emerge on the benefits of early and simultaneous administration of blood products in pre-specified ratios. The findings from this study provide a previously unreported baseline from which the potential impact of changes to service provision and/or treatment practice can begin to be evaluated. Further studies are still required to determine the full costs of post-discharge care requirements, which are also likely to be substantial.

**Electronic supplementary material:**

The online version of this article (doi:10.1186/s13054-015-0987-5) contains supplementary material, which is available to authorized users.

## Introduction

Trauma is a leading cause of death and disability around the world. An estimated 15 % of major trauma patients present to hospital with severe bleeding, yet 40 % of all major trauma deaths can be attributed to haemorrhage [1]. Bleeding patients are therefore a clinically significant subgroup of the trauma population and have been the focus of recent research on trauma physiology, haemostatic agents, near-patient coagulation testing, damage control resuscitation, and consolidating best-practice management [1–8]. The costs to the healthcare provider of treating severely bleeding trauma patients are likely to be sizeable but have received less attention. Despite calls for better information on these costs, and whilst estimates for blunt and penetrating major trauma in England and Wales have been published, the cost burden to the National Health Service (NHS) of treating severe bleeding in trauma has yet to be fully quantified [9–11].

Detailed knowledge of the costs of severe bleeding in trauma is essential for a range of stakeholders. For health service managers, understanding the key cost drivers can help with the development of targeted cost reduction strategies. For health economists, such data can be used as inputs into studies evaluating the cost-effectiveness of new bleeding cessation interventions [12]. For policy planners, the nation-wide cost burden of severe bleeding in trauma can be compared with that of other conditions, and potential future costs, such as those resulting from an aging population, can be modelled.

As part of a broader prospective cohort study investigating the incidence, treatment, and outcomes of severe traumatic bleeding, a detailed patient-level assessment of the resource use and costs required to treat severely bleeding major trauma patients arriving at NHS hospitals was performed. Specific objectives of the costing component of the study that is presented in this paper were to: 1) generate estimates of patient-level resource use and healthcare costs, 2) to explore whether costs vary across particular clinical subgroups, and 3) to scale up patient-level figures to a national level for England using Hospital Episode Statistics (HES) data.

## Methods

The work was conducted in collaboration with the Trauma Audit Research Network (TARN), the independent monitor of trauma care in England and Wales [13]. TARN collects information from hospitals in England and Wales on all presenting major trauma patients (those with a hospital length of stay of 72 hours or more, and/or requiring intensive or high dependency care, and/or where a death occurred in hospital). Data coordinators at hospitals record detailed patient-level information on treatments and care received, and mortality at hospital discharge and at 30 days. All data are anonymised and entered into TARN’s Electronic Data Collection and Reporting (EDCR) system.

Twenty two hospitals (21 in England and one in Wales) submitting data to TARN were recruited to the prospective study. The general methodology and main study outcomes have been submitted for publication, and are briefly described as follows [14]. Study hospitals were selected to ensure adequate geographical dispersion and a balanced representation of large multi-specialty trauma centres (12 centres) and medium-sized hospitals with trauma units (10 centres). Following Research and Development approvals, the study enrolled consecutive patients with severe traumatic bleeding at the different hospitals, where severe traumatic bleeding was defined by a transfusion need for four units or more of red cells and activation of a massive haemorrhage protocol (MHP). A MHP supports the immediate and simultaneous administration of different blood products in pre-specified ratios, and its activation serves as an indicator that the degree of bleeding is considered significant enough to warrant multi-component transfusion therapy for its attempted control. Only index hospital admissions were sought, and so patients transferred into a study hospital after receiving treatment elsewhere were excluded. Blood component transfusion data were not routinely collected by TARN, and so for the purposes of the study, additional fields for units of blood components issued, transfused, and wasted, and any transfusion-related complications reported were added to the EDCR system. The follow-up period for patient mortality was extended to 12 months post-injury. Study data collection took place between 1 April 2009 and 31 March 2011. The 12-month follow-up period for the last patient entering the study ended on 31 March 2012.

The National Research Ethics Committee South Central Oxford B gave full ethical approval (approval number: 09/H0605/58) for the study. TARN already had Patient Information Advisory Group approval for data collection to include individual patient’s NHS number, age, gender, ethnicity, incident postcode, and sector postcode for home, and so no further permissions were required. A separate application to the National Information Governance Board Ethics and Confidentiality Committee and NHS Medical Register Information Centre allowed the processing of patient identifiable information without consent and the collection of one-year survival and mortality data for participants.

Overall aims of the study as a whole were to: describe the characteristics of patients with major traumatic injuries and bleeding admitted to trauma-receiving emergency departments (ED); develop an estimate of the national incidence of patients requiring four or more units of packed red blood cells (PRBCs); report on the use of PRBCs and fresh frozen plasma (FFP); estimate NHS treatment costs; and describe mortality during 24 hours, at 30 days, and one year.

### Resource use data

**Fig. 1:**
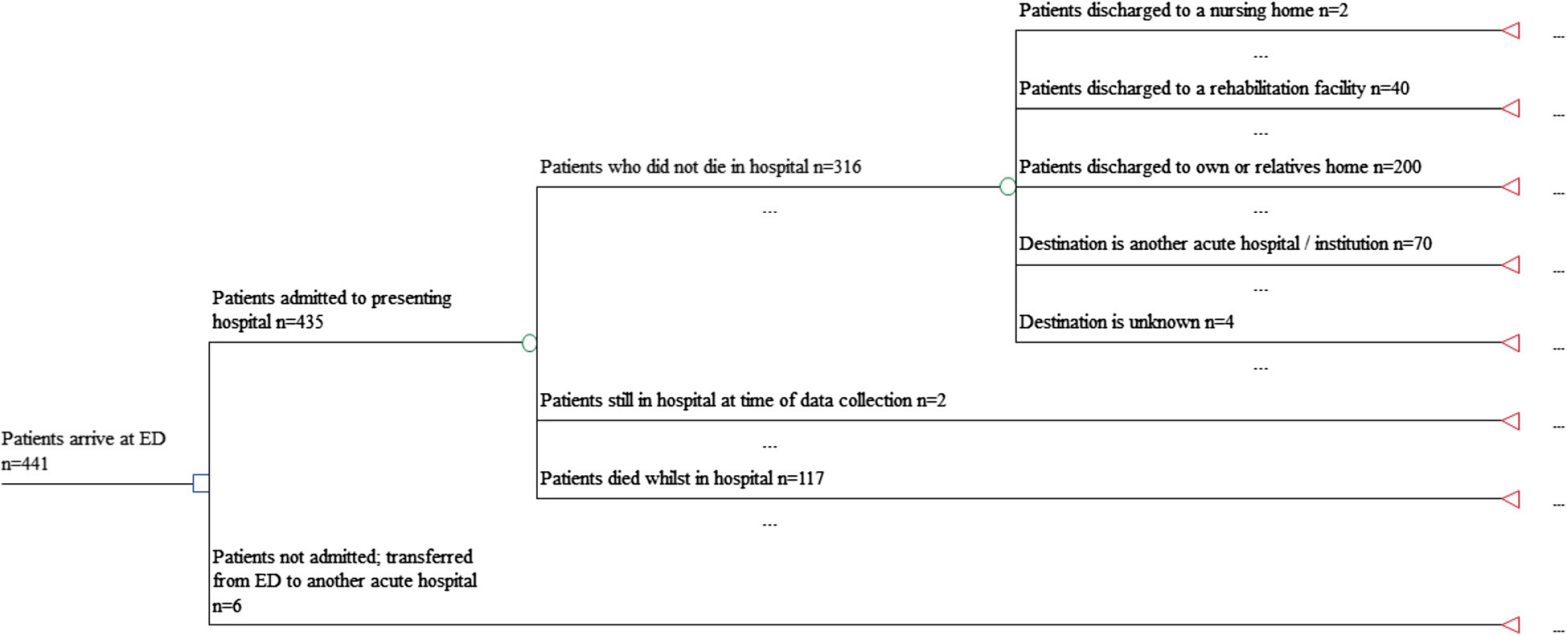
Patient pathways following presentation at an Emergency Department (ED)

Figure 1 illustrates the care pathways taken by patients in the study cohort. For each patient, data were available on mode of emergency transport to hospital, duration spent in the ED, and Accident and Emergency (A&E) consultant and/or trauma team attendances.

From ED presentation until hospital discharge, transfer, or death, data on haematology tests (full blood counts and clotting investigations) and imaging investigations (X-ray, computed tomography, magnetic resonance imaging, and ultrasound) were collected, along with neurologist, general surgeon, and cardiologist consultations, and the use of pro-coagulant medications (recombinant Factor VIIa and Vitamin K).

Information was collected on the number of units of blood components (PRBCs, FFP, platelets, and cryoprecipitate) and pro-haemostatic concentrates (such as prothrombin complex concentrate (Beriplex)) administered up to 24 hours following presentation, and from hour 25 to 30 days (or discharge, transfer, or death if earlier). Blood banks provided data on units of each blood component issued to a patient that were then wasted, and on any transfusion-related complications. Volumes of intravenous (IV) fluids given up to 24 hours following presentation were also recorded.

For patients admitted to the hospital from the ED, time (hours) spent in theatre, intensive care units (ICU), and on general wards was documented. Inpatient days spent receiving ventilation were also recorded. A small number of patients were transferred directly from the ED to another acute hospital, however no inpatient resource use data were collected from receiving hospitals. Similarly for patients surviving the index hospital admission who were then discharged to another acute hospital or institution, acute care was considered to be ongoing but further information was not collected. Patients discharged to nursing homes and rehabilitation units entered a post-acute phase of care, but the duration of time spent in these facilities was unknown.

The occurrence and duration of re-admissions to initial treating hospitals were recorded. It was assumed that re-admission checks were conducted frequently for each study patient, and as the final study dataset was received from TARN after the time since trauma injury exceeded 12 months for all patients, all re-admissions to initial treating hospitals over 12 months were recorded.

### Costs

Patient-level costing was conducted from the perspective of the UK NHS, with costs expressed in 2012 to 2013 UK Pound Sterling. Table 1 shows the unit costs used to value the resource use data collected, and Additional file 1 provides supplementary detailed information on the costing methods used. Unit costs from national sources were used in the first instance, supplemented with local costs when necessary.

**Table 1:** Unit costs used to value TARN resource use data

Resource	Unit Cost UK £ 2012 to 2013	Source
ED, theatre, and ward stays		
Ambulance transfer to hospital	£230.00	NHS Reference Costs 2012 to 2013 - code ASS02 [15]
Helicopter transfer to hospital	£1,844.00	http://www.yorkshireairambulance.org.uk/files/file/content/YAAreportaccounts_endmarch2012.pdf
ED attendance	£319.00	NHS Reference Costs 2012 to 2013 - weighted average code VB01Z (AE worksheet) [15]
A&E Consultant contact	£121.00	NHS Reference Costs 2012 to 2013 - code 180 (Total Outpatient Attendances worksheet) [15]
Trauma team (per hour)	£477.54	Expert opinion on staffing/Unit Costs of Health and Social Care 2013, PSSRU [17]
Theatre time (per hour)	£881.00	ISD Scotland Theatre 2013 - Costs - Detailed Tables (Theatres). Sheet R142X, A&E [26]
Intensive care bed day	£619.00	NHS Reference Costs 2012 to 2013 - code XC07Z (CC worksheet) [15]
Non-intensive care bed day	£236.00	NHS Reference Costs 2012 to 2013 - weighted average code HA96Z (NEI_L_XS worksheet) [15]
Incremental daily cost of ventilation	£861.84	Dasta *et al*. 2005 inflated using Unit Costs of Health and Social Care 2013, PSSRU [16, 17]
Haematology and clotting tests		
Full blood count	£3.38	Finance Department, TARN participating hospital
Fibrinogen	£4.85	Finance Department, TARN participating hospital
APTT	£3.38	Finance Department, TARN participating hospital
PT	£3.38	Finance Department, TARN participating hospital
INR	£3.38	Finance Department, TARN participating hospital
APTTR	£3.38	Finance Department, TARN participating hospital
Imaging investigations		
CT scan	£67.57	NHS Reference Costs 2012 to 2013 - codes RA08A, RA09A, RA10Z-RA14Z (service code 110) (DIAGIM worksheet) [15]
X-ray	£40.00	Finance Department, TARN participating hospital
Ultrasound	£60.95	NHS Reference Costs 2012 to 2013 - codes RA23Z, RA24Z, RA26Z (service code 110) (DIAGIM worksheet) [15]
MRI	£78.73	NHS Reference Costs 2012 to 2013 - codes RA01A, RA04Z (service code 110) (DIAGIM worksheet) [15]
Blood components (per unit)		
PRBCs	£123.31	NHS Blood and Transplant Price List 2012 to 2013
FFP	£27.46	NHS Blood and Transplant Price List 2012 to 2013
Platelets	£209.30	NHS Blood and Transplant Price List 2012 to 2013
Cryoprecipitate	£189.19	NHS Blood and Transplant Price List 2012 to 2013
Beriplex	£200.00	Finance Department, TARN participating hospital
Transfusion laboratory issue cost	£2.00	Details available from the authors upon request
Fluids (500 mL)		
Dextrose	£1.07	Finance Department, TARN participating hospital
Colloids	£2.64	Finance Department, TARN participating hospital
Crystalloids	£0.71	Finance Department, TARN participating hospital
Polygelatine	£2.14	World Health Organisation Technical Report [27]
Starch	£8.00	Finance Department, TARN participating hospital
Hypertonic saline	£3.56	Finance Department, TARN participating hospital
Albumin	£35.00	Finance Department, TARN participating hospital
Hartmann’s	£0.89	Finance Department, TARN participating hospital
Consultant contacts		
Neurologist	£178.00	NHS Reference Costs 2012 to 2013 - code 400 [15]
Cardiologist	£143.00	NHS Reference Costs 2012 to 2013 - code 320 [15]
General surgeon	£133.00	NHS Reference Costs 2012 to 2013 - code 100 [15]
Pro-coagulants		
Factor VIIa	£994.94	Finance Department, TARN participating hospital
Vitamin K	£0.38	British National Formulary 2012 [28]
Post-hospital discharge		
Rehabilitation (per episode)	£2,758.00	Unit Costs of Health and Social Care 2013, PSSRU, Section 1.6 [17]
Nursing home (per day)	£143.14	Unit Costs of Health and Social Care 2013, PSSRU, Section 1.3 [17]
Re-admissions to original hospital		
Intensive care bed day	£619.00	NHS Reference Costs 2012 to 2013 - code XC07Z (CC worksheet) [15]
Non-intensive care bed day	£236.00	NHS Reference Costs 2012 to 2013 - weighted average code HA96Z (NEI_L_XS worksheet) [15]

*AE* Accident and Emergency Services, *A&E* Accident and Emergency, *APTT* Activated Partial Thromboplastin Time, *APTTR* Activated Partial Thromboplastin Time Ratio, *CC* Critical Care, *CT* Computed Tomography, *DIAGIM* Diagnostic Imaging, *ED* Emergency Department, *FFP* Fresh Frozen Plasma, *INR* International Normalised Ratio, *ISD* Information Services Division, *mL* Millilitres, *MRI* Magnetic Resonance Imaging, *NEI_L_XS* Non-Elective Inpatient (Long Stay) Excess Bed Days, *PRBCs* Packed Red Blood Cells, *PSSRU* Personal Social Services Research Unit, *PT* Prothrombin Time, *TARN* Trauma Audit Research Network

Emergency transport to the ED was costed. All patients were assigned the cost of an ED visit, and an A&E consultant contact when recorded. Time spent in the ED was used when costing the intervention of the trauma team, which included general and orthopaedic surgeons (see Additional file 1 for a full list team members). The team was assumed to be present for up to a maximum of one hour into a patient’s time in the ED, after which patients were assumed to be stable, receiving intensive care in resuscitation, and awaiting the availability of an inpatient bed. This time was costed using the appropriate proportion of an ICU bed day cost [15].

As IV fluids are supplied in 500 mL and 1 L bags and blood components as unit bags, both were costed to allow for wastage (for example if two and a half units of PRBCs were transfused, a cost of three units was assigned). Added to each unit of blood component transfused and wasted was the estimated cost of hospital transfusion laboratory staff involved in component issuing.

Time in theatre was costed to allow for the running cost of the theatre (including surgeons and nursing staff). Hours in ICU were costed using an ICU bed day cost, assuming no organ support [15]. The incremental daily cost of ventilation was taken from the published literature (Table 1) and applied to the number of ventilated days recorded for each patient [16]. Non-ICU ward days were costed using a national excess bed day cost averaged across multiple trauma episodes from the NHS Reference Costs [15].

An ambulance cost was assigned to patients transferred or discharged to another acute hospital or institution. A series of assumptions were made about the duration and intensity of acute care (ICU, ventilation, and ward) provided at receiving hospitals (see Additional file 1 for full details).

It was assumed that patients discharged to a nursing home would have been resident for the remainder of their 12-month study period, and this time was costed [17]. Discharges to rehabilitation units were costed based upon the published literature, which suggests an average length of stay of 33 days at a cost of £2,758 (Table 1) [17]. ICU and ward stay data were documented for re-admissions to the initial treating hospital in the 12 months following injury, and were costed using the appropriate bed day costs.

### Data analysis

A total of 12 % of resource use data were missing (a breakdown by variable is shown in columns two and three of Additional file 1: Table A1). Exploration of the data suggested patients with missing data were more likely to have been treated at large major trauma centres than at medium-sized trauma units, and were also less likely to have been discharged to another hospital or institution. Data were assumed to be missing at random, and multiple imputation (MI) with chained regression equations (which included hospital and discharge location as predictors) was used to impute 20 values for each missing data point, essentially creating 20 different datasets (for full details see Additional file 1) [18].

Categorical variables were summarised as frequencies and percentages, and continuous variables using means and standard errors (SE). Parametric 95 % confidence intervals (Cis) are presented for total cost estimates. Rubin’s rule was used when summarising data across the five datasets created using MI [19]. When calculating measures of precision, this approach allows the variability both within and between imputed datasets to be accounted for. All analyses were conducted in Stata Version 12.1 (StataCorp, College Station, Texas, USA).

### One-way sensitivity analysis

A number of potential sources of uncertainty exist in the analysis. The cost of an unventilated bed day on ICU was increased from £619 to £1,200 (sensitivity analysis (SA)1). It was assumed that half of the 174 inpatients for whom a theatre admission was possible but unrecorded, did undergo surgery, but that these data had been missing (SA2). For these patients, theatre duration was inferred conditional upon age, sex, type of injury, and Injury Severity Score (ISS). It was assumed that all 92 patients for whom no trauma team attendance was recorded, were treated by the team in the ED but that these data had been unrecorded (SA3). Finally, re-admissions to other (non-study) hospitals are likely to have occurred but were not captured by the study. The impact of 100 % and 200 % increases in the number of re-admissions observed over the study period was also assessed (SA4 and SA5).

### Subgroup analysis

Subgroup analyses were not specified in the study protocol, however costs were summarised for a number of clinically relevant groups, including patients classified as suffering major haemorrhage (receiving four to nine units of PRBCs in 24 hours), and those suffering massive haemorrhage (receiving 10 or more units of PRBCs in 24 hours). Costs were also estimated according to injury severity (an ISS score of 15 or more indicative of more severe injury), and injury type (blunt versus penetrating). Mean cost differences, parametric 95 % CIs, and t-tests were used when comparing costs between subgroups. A *P* value of less than 0.05 was considered to be statistically significant. For completeness, in-hospital mortality for subgroups was also reported and compared. The *Χ*^2^ test was used when comparing proportions of deaths in each subgroup, and a *P* value of less than 0.05 was again considered to be statistically significant.

### National cost estimates

The HES database provided information on the number of patients (by gender and for 10-year age bands) who presented to all hospitals in England over the study period, and who would have met the TARN eligibility criteria for major trauma [20]. Using all TARN patients (bleeding and non-bleeding) from the 22 study centres, estimates were then made (again by gender and 10-year age bands) of the proportion of major traumas that are associated with severe bleeding (meeting the eligibility criteria of this study). These proportions were assumed to be nationally representative, and were applied (following adjustment for potential under-reporting to TARN) to the HES data to estimate the total number of major trauma cases with severe haemorrhage in England. This same approach was also used for the subgroup of patients classified as having massive haemorrhage (receiving 10 or more PRBCs units in the first 24 hours following presentation).

Mean total healthcare costs per patient estimated by this study for patients with severe haemorrhage treated at English hospitals only (n = 414 out of 447), and for the subgroup with massive haemorrhage, were summarised using the same gender and 10-year age categories and multiplied by the appropriate estimated national case numbers to give an estimate of the costs of severe and massive haemorrhage in major trauma patients in England. Uncertainty around these estimates was explored by increasing and decreasing the estimated national case numbers by 5 %, and also by increasing and decreasing the mean total cost estimates for each age and gender subgroup by 10 % and 20 % respectively.

## Results

A total of 441 patients met the study entry criteria. Almost three quarters of patients were male, and the mean age of the cohort was 41.9 years. Results showed that 80 % of patients suffered blunt injuries and the mean ISS for the cohort was 29.5. Mortality results showed that 117 out of 441 (26.5 %) patients died whilst in hospital, 120 out of 436 (27.5 %) died within 30 days, and 145 out of 403 (36.0 %) died within 12 months. Figure 1 shows patient pathways for these patients and Table 2 shows demographics and injury characteristics.

**Table 2:** Demographic, injury, and mortality data

Patient/Injury characteristics	Cohort
	n = 441
Gender - male (%)	73.7 %
Age - years, mean (SD)	41.9 (19.8)
Injury type	
Blunt (%)	79.9 %
Penetrating (%)	20.2 %
Injury Severity Score - mean (SD)	29.5 (15.8)
Sites of injury (%)	
Head	41.9 %
Chest	67.0 %
Abdomen	42.3 %
Spine	31.4 %
Lower limb	63.8 %
Upper limb	48.2 %
Burns	0.9 %
Other body surface	5.0 %
In-hospital mortality, n (%)	117/441 (26.5 %)
Mortality at 30 days, n (%)	120/436^a^ (27.5 %)
Mortality at 12 months, n (%)	145/403^b^ (36.0 %)

^a^Survival status was missing for five patients at 30 days

^b^Survival status was missing for 38 patients at 12 months

### Hospital inpatient resource use and costs

Table 3 shows a detailed breakdown of the inpatient resource use and mean total cost per patient. Mean (SE) time in the ED was 2.96 (0.11) hours, and more than half (236 out of 441, 53 %) of patients had at least one admission to theatre. Time in ICU, non-ICU wards, and on ventilation averaged 7.93 (0.54) days, 19.58 (1.21) days, and 3.23 (0.41) days, respectively. During the first 24 hours patients received an average of 9.87 (0.38) units of PRBCs and 4.92 (0.28) units of FFP. No transfusion-related complications were reported.

**Table 3:** Mean (SE) inpatient resource use and costs per patient

Resource	Frequency (%) or mean (SE) n = 441	Per patient cost mean (SE) n = 441
Pre-hospital transport^a^		
Ambulance transport to hospital	272/441 (62 %)	--
Helicopter transport to hospital	139/441 (31 %)	--
Ambulance and helicopter transport	26/441 (6 %)	--
Total cost of emergency transport	--	£842.76 (£39.52)
Emergency Department		
ED admission	1.00 (0.00)	£319.00 (£0.00)
Time in ED (hours)	2.96 (0.11)	£72.02 (£3.02)
Seen by A&E Consultant	354/441 (80 %)	£97.10 (£2.30)
Attended by trauma team	349/441 (79 %)	£339.76 (£9.40)
Theatre admissions		
Theatre admission recorded		
0 theatre admissions	205/441 (47 %)	--
1 theatre admission	158/441 (36 %)	--
2 theatre admissions	37/441 (8 %)	--
3+ theatre admissions	41/441 (9 %)	--
Total time in theatre (hours)	3.58 (0.24)	£3,154.53 (£214.02)
Intensive care		
Time in intensive care (days)^b^	7.93 (0.54)	£4,895.20 (£333.31)
Ward stay		
Time in non-ICU wards (days)^b^	19.58 (1.21)	£4,619.05 (£286.19)
Ventilated inpatient days		
Time on ventilation (days)^b^	3.23 (0.41)	£2,779.80 (£355.37)
Haematology and clotting tests		
Full blood count	1.76 (0.07)	£5.95 (£0.23)
Fibrinogen	0.81 (0.08)	£3.94 (£0.41)
APTT	1.05 (0.09)	£3.55 (£0.30)
PT	1.32 (0.08)	£4.48 (£0.27)
INR	0.51 (0.07)	£1.72 (£0.23)
APTTR	0.29 (0.06)	£0.99 (£0.19)
Imaging investigations		
CT scan	1.51 (0.07)	£101.94 (£4.79)
X-ray	1.72 (0.11)	£68.71 (£4.59)
Ultrasound	0.08 (0.01)	£4.85 (£0.86)
MRI	0.06 (0.01)	£5.03 (£1.08)
Blood components transfused in the first 24 hours		
PRBCs	9.87 (0.38)	£1,237.19 (£48.07)
FFP	4.92 (0.28)	£145.03 (£8.24)
Platelets	0.96 (0.08)	£204.59 (£16.47)
Cryoprecipitate	0.74 (0.08)	£143.07 (£15.86)
Beriplex	0 (0)	£0.00 (£0.00)
Blood components transfused after 24 hours		
PRBCs	2.61 (0.24)	£326.77 (£29.99)
FFP	0.74 (0.14)	£21.84 (£4.24)
Platelets	0.24 (0.04)	£50.79 (£8.83)
Cryoprecipitate	0.12 (0.05)	£23.84 (£8.85)
Beriplex	0.01 (0.01)	£2.29 (£2.29)
Blood components issued then wasted		
PRBCs	0.74 (0.14)	£92.35 (£17.75)
FFP	0.84 (0.11)	£24.75 (£3.19)
Platelets	0.11 (0.03)	£23.93 (£5.89)
Cryoprecipitate	0.34 (0.06)	£65.18 (£11.99)
Fluids (mLs) given in the first 24 hours		
Dextrose	47.74 (13.07)	£0.10 (£0.03)
Colloids	564.05 (61.49)	£3.02 (£0.33)
Crystalloids	1595.74 (108.33)	£2.33 (£0.15)
Polygelatine	80.50 (19.97)	£0.34 (£0.09)
Starch	4.17 (2.87)	£0.12 (£0.06)
Hypertonic saline	381.29 (51.37)	£2.82 (£0.38)
Albumin	8.62 (6.12)	£0.71 (£0.44)
Hartmann’s	1141.43 (95.80)	£2.05 (£0.17)
Consultant contacts		
Neurologist	17/441 (4 %)	£6.94 (£1.65)
Cardiologist	12/441 (3 %)	£3.96 (£1.13)
General surgeon	42/441 (10 %)	£12.71 (£1.87)
Pro-coagulants		
Factor VIIa	6/441 (1 %)	£13.54 (£5.49)
Vitamin K	1/441 (<1 %)	£0.01 (£0.01)
Ambulance for patients transferred/discharged to another acute hospital/institution	76/441 (17 %)	£39.64 (£4.14)
Total inpatient healthcare costs	--	£19,770.29 (£810.44) (95 % CI: £18,176.83 to £21,363.74)

*A&E* Accident and Emergency, *APTT* Activated Partial Thromboplastin Time, *APTTR* Activated Partial Thromboplastin Time Ratio, *CT* Computed Tomography, *ED* Emergency Department, *FFP* Fresh Frozen Plasma, *INR* International Normalised Ratio, *ICU* Intensive Care Unit, *mLs* Millilitres, *MRI* Magnetic Resonance Imaging, *PRBCs* Packed Red Blood Cells, *PT* Prothrombin Time, *SE* Standard Error

^a^Four patients arrived via own transport

^b^Includes estimates of ICU/ward stay/ventilation in acute hospitals to which patients were discharged or transferred

The mean total inpatient cost was £19,770 (SE: £810; 95 % CI: £18,177 to £21,364), of which 62 % was attributable to ventilation, ICU, and ward stays, and a further 16 % to surgery. Blood component costs accounted for 12 % of the total inpatient costs at £2,362 (SE: £115); 70 % of this cost (£1,656) was attributable to PRBCs and 8 % (£191) to FFP.

### Post-acute care resource use and costs

Out of 316 patients, 40 (13 %) surviving the inpatient admission at the presenting hospital were discharged to a rehabilitation unit, and just two (0.6 %) were discharged to a nursing home (Fig. 1). Table 4 shows the cost of this care averaged across the whole cohort. Mean total costs to 12 months were £20,591 (SE: £848; 95 % CI: £18,924 to £22,257) per patient (Table 4).

**Table 4:** Healthcare costs (2012 to 2013 £UK) up to 12 months for the whole cohort

Phase of care	Mean (SE) Cost (n = 441)
Acute hospital care	£19,770.29 (£810.44)
Immediate post-discharge care	
Nursing home	£205.18 (£145.18)
Rehabilitation unit	£250.16 (£37.76)
Hospital re-admissions	£364.88 (£96.88)
Total costs	£20,590.51 (£847.77) (95 % CI: £18,923.72 to £22,257.30)

### Re-admissions to original treating hospital at 12 months

A total of 125 patients died during their acute period of care (117 during admission to the presenting hospital, and eight soon after being transferred or discharged to another acute hospital). Amongst the remaining 316 patients, there were 26 (8 %) re-admissions in the 12 months following trauma. Mean re-admission ICU stay was 0.8 (SE: 0.8) days and mean ward stay was 23.8 (SE: 4.41) days. The mean cost per re-admission recorded was £6,095 (SE: £1,189). Table 4 shows re-admission costs averaged across the whole cohort.

### One-way sensitivity analysis

**Fig. 2:**
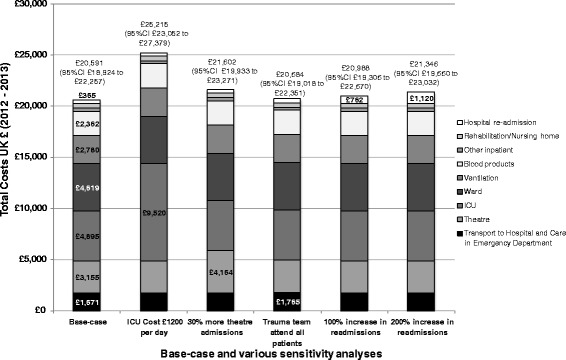
Stacked bar chart showing key cost categories and mean (95 % CI) total cost per patient for various one-way sensitivity analyses

Figure 2 shows total healthcare costs and a breakdown of key costs by category for the various sensitivity analyses. Results were most sensitive to a doubling of the daily cost on the ICU (SA1); the mean overall total cost per patient increased from £20,591 (95 % CI: £18,924 to £22,257) to £25,215 (95 % CI: £23,052 to £27,379).

### Subgroup analysis

Table 5 details costs and mortality for each subgroup analysis performed. Massive haemorrhage patients cost, on average £3,482 more to treat than major haemorrhage patients (95 % CI: −£74 to £7,037, *P* = 0.06). Blood component costs were around three times higher (£4,174 versus £1,455: difference of £2,719 (95 % CI: £2,320 to £3,118, *P* <0.01)), and in-hospital mortality was almost twice that observed for major haemorrhage patients (proportion 0.38 (56 out of 147) versus proportion 0.21 (61 out of 294), *P* <0.01).

**Table 5:** Mean (SE) costs by category, total costs (2012 to 2013 £UK) and mortality, by subgroup

	Whole cohort	Subgroup by blood volume		Subgroup by ISS		Subgroup by injury type	
	n = 441	4 to 9 PRBCs n = 294	≥10 PRBCs n = 147	ISS <15 n = 85	ISS ≥15 n = 356	Blunt n = 352	Penetrating n = 89
Transport to ED cost	£1,671 (£42)	£1,670 (£52)	£1,671 (£74)	£1,420 (£93)	£1,730 (£47)	£1,810 (£47)	£1,121 (£64)
Theatre cost	£3,155 (£214)	£3,159 (£244)	£3,146 (£419)	£3,426 (£443)	£3,090 (£243)	£3,194 (£258)	£2,999 (£294)
ICU cost	£4,895 (£333)	£4,793 (£403)	£5,099 (£593)	£2,685 (£455)	£5,423 (£394)	£5,426 (£399)	£2,795 (£420)
Ward cost	£4,619 (£286)	£4,937 (£354)	£3,983 (£483)	£3,897 (£435)	£4,791 (£339)	£5,139 (£340)	£2,564 (£383)
Ventilation cost	£2,780 (£355)	£2,285 (£375)	£3,769 (£801)	£1,573 (£486)	£3,068 (£421)	£3,195 (£424)	£1,139 (£267)
Blood component cost	£2,362 (£115)	£1,455 (£64)	£4,174 (£261)	£1,615 (£146)	£2,540 (£136)	£2,270 (£105)	£2,723 (£390)
Other inpatient costs	£289 (£12)	£278 (£13)	£312 (£26)	£169 (£16)	£318 (£14)	£322 (£14)	£159 (£20)
Post-acute care cost	£455 (£149)	£570 (£221)	£225 (£62)	£130 (£64)	£533 (£184)	£563 (£186)	£31 (£31)
Re-admission cost	£365 (£97)	£281 (£94)	£533 (£222)	£318 (£179)	£376 (£112)	£452 (£121)	£21 (£38)
Mean (95 % CI) total cost	£20,591 (£18,924 to £22,257)	£19,429 (£17,567 to £21,293)	£22,912 (£19,525 to £26,298)	£15,233 (£12,970 to £17,495)	£21,870 (£19,895 to £23,845)	£22,370 (£20,383 to £24,358)	£13,552 (£11,748 to £15,356)
In-hospital mortality - n (prop)	117/441 (0.27)	61/294 (0.21)	56/147 (0.38)	5/85 (0.06)	112/356 (0.31)	100/352 (0.28)	17/89 (0.19)
12-month mortality - n (prop)	145/403 (0.36)	79/263 (0.30)	66/140 (0.47)	11/74 (0.15)	134/329 (0.41)	122/324 (0.38)	23/79 (0.29)

*CI* Confidence Interval, *ED* Emergency Department, *ICU* Intensive Care Unit, *ISS* Injury Severity Score, *PRBCs* Packed Red Blood Cells, *Prop* Proportion, *SE* Standard Error

Severely injured patients (ISS ≥15) were over 40 % more costly to treat than patients with less severe injuries (£21,870 versus £15,233, mean total cost difference £6,637 (95 % CI: £2522 to £10,753, *P* <0.01) and appeared five times more likely to die in hospital (proportion 0.31 (112 out of 356) versus proportion 0.06 (5 out of 85), *P* <0.01) than less injured patients.

Patients with penetrating injuries incurred average costs of £13,552, however the cost of treating blunt injuries was higher at £22,370 (mean total cost difference £8,818 (95 % CI: £4,844 to £12,793, *P* <0.01)). There was a trend towards higher mortality in blunt trauma patients (proportion 0.28 (100 out of 352) versus proportion 0.19 (17 out of 89), *P* = 0.05).

### National cost estimates

Of the 49,859 major trauma cases presenting to hospitals each year in England, 7,783 (15.6 %) were estimated to have severe haemorrhage (Table 6). Associated costs were estimated to be £148,293,657. Within this figure, 32 % of patients were estimated to have had massive haemorrhage, for which the estimated cost was £56,406,200.

**Table 6:** Estimated total annual costs for severely bleeding trauma patients in England and for the subgroup with massive haemorrhage

	Annual number of major trauma cases with severe bleeding in England^a^	Estimated mean cost per case (number of cases estimate based on)^b^	Annual total cost of major trauma cases with severe bleeding in England	Annual number of major trauma cases with massive haemorrhage in England^a^	Estimated mean cost per case (number of cases estimate based on)^b^	Annual total cost of major trauma cases with massive haemorrhage in England
Age 16 to 24						
Male	556	£19,176 (n = 71)	£10,661,856	204	£17,173 (n = 25)	£3,503,292
Female	236	£25,574 (n = 26)	£6,035,464	109	£20,225 (n = 12)	£2,204,525
Age 25 to 34						
Male	634	£20,167 (n = 67)	£12,785,878	210	£28,212 (n = 24)	£5,924,520
Female	212	£17,631 (n = 19)	£3,737,772	48	£20,728 (n = 3)	£994,944
Age 35 to 44						
Male	679	£20,992 (n = 56)	£14,253,568	259	£23,357 (n = 21)	£6,049,463
Female	361	£20,502 (n = 15)	£7,401,222	116	£15,568 (n = 5)	£1,805,888
Age 45 to 54						
Male	615	£20,496 (n = 46)	£12,605,040	178	£23,823 (n = 14)	£4,240,494
Female	377	£20,469 (n = 16)	£7,716,813	75	£15,519 (n = 4)	£1,163,925
Age 55 to 64						
Male	650	£22,308 (n = 32)	£14,500,200	235	£27,875 (n = 11)	£6,550,625
Female	419	£18,378 (n = 15)	£7,700,382	133	£25,026 (n = 6)	£3,328,458
Age 65+						
Male	1,641	£14,479 (n = 28)	£23,760,039	435	£11,005 (n = 7)	£4,787,175
Female	1,403	£19,341 (n = 23)	£27,135,423	489	£32,419 (n = 5)	£15,852,891
Totals	7,783	--	£148,293,657	2,491	--	£56,406,200

^a^Estimates of annual numbers of cases based on HES and TARN data

^b^Mean cost estimates based upon analysis of TARN data reported in this paper

Assuming a 5 % increase and decrease in national case figures altered the total severe haemorrhage cost to £155,708,340 and £140,878,974, respectively, and the massive haemorrhage costs to £59,226,510 and £53,585,890, respectively. A 10 % increase and decrease in mean total costs for each age and gender patient subgroup in Table 6 altered the total severe haemorrhage cost to £163,123,023 and £133,464,291, respectively, and the massive haemorrhage costs to £62,046,820 and £50,765,580, respectively. With a 20 % increase and decrease, corresponding figures were £177,952,388 and £118,634,926 for severe haemorrhage, and £67,687,440 and £45,124,960 for massive haemorrhage.

## Discussion

This study has generated estimates of healthcare costs associated with major trauma patients with severe bleeding. Strengths of the study and the cost estimates are data collection across a broad range of hospitals and trauma centres, likely to be representative of national practice. The mean cost per patient for immediate in-hospital care was £19,770 (SE: £810), with much of this cost attributable to surgery, intensive care, ventilation, and ward stay. Blood components accounted for 12 % of inpatient costs at £2,362 (SE: £115) per patient. Sensitivity analyses showed cost results were most sensitive to changes in the ICU bed day cost.

Scaling up data to a national level has suggested around 7,800 severely bleeding adult major trauma patients present to hospitals in England each year, at a total cost to treating NHS hospitals of £148,300,000. Sensitivity analyses showed there to be some uncertainty around this estimate, however costs generally remained high. Bleeding trauma patients are therefore a small yet high cost group compared to other types of bleeding patients. By comparison, a recent study of healthcare costs in acute upper gastrointestinal haemorrhage suggested around 57,000 patient presentations per year in the UK, costing a total of £155,500,000 in initial hospital care [21]. Whilst clinicians and policy makers may find the figures in this paper helpful, it should be acknowledged that they are representative of a single country only, and furthermore a country, which in the NHS, has a unique healthcare financing arrangement and system. The detailed breakdown of resource use will allow readers to determine whether the healthcare provided is representative of their own settings, however caution should still be exercised when generalising the findings beyond the NHS. Additionally, and as with any costing study, the nature of the data presented, that is the point estimates isolated from contextual variability that may exist, should also be borne in mind.

Confidence intervals around total costs in this study were estimated using the Central Limit Theorem (CLT) approach, which relies on sample mean costs being normally distributed. The suitability of CLT when data are skewed (which cost data invariably are) has been questioned, and the bootstrap approach has been proposed as an alternative. Recent studies have suggested however, that even with high levels of skewness, provided the study sample is moderate to large (n >50), CLT can still provide accurate estimates of the standard error and accompanying confidence intervals [22]. With a study sample of 441, this approach to confidence interval estimation was considered appropriate.

As few studies have comprehensively assessed the costs associated with bleeding trauma patients, it is difficult to set the estimates presented here within the context of existing research. In 2007, Morris *et al*. presented estimates of the costs to the NHS of treating bleeding blunt trauma patients as part of a trial-based cost-effectiveness analysis of recombinant factor seven A versus placebo, and reported average acute care costs of £26,256 per patient in the placebo arm [7]. Patients in that study however, all suffered extreme haemorrhage (receiving at least six units of PRBCs within just four hours), making them different to the cohort studied here. The subgroup of patients within this study suffering massive haemorrhage (receiving 10 or more units of PRBCs in 24 hours) perhaps provide a more appropriate (albeit still imperfect) comparator, and have relatively similar costs at £22,912 per patient (Table 5).

Data from this study suggests that over 40 % (3,044 out of 7,783) of major trauma patients presenting with severe bleeding are likely to be aged 65 and over. With the percentage of the population in England in this age category projected to increase by almost 22 %, from 17.8 % in 2014 to 21.5 % in 2030, the total number of bleeding trauma patients in England could reach almost 8,450 by 2030 [23]. With high estimated average costs per additional case (£16,720 per patient aged 65 years and above), this implies significant additional costs of £11,200,000 for in-hospital treatment alone. Future public health initiatives in trauma prevention programmes might be targeted at the elderly, to offer a potential means of reducing this future cost burden.

The resource use and cost estimates generated by this study should prove useful as a baseline for a variety of stakeholders. Scaling up blood component wastage data from this study (Table 3) to national levels, suggests that approximately 5,759 units of the PRBCs and 6,538 units of the FFP issued to severely bleeding trauma patients annually are wasted, at costs of £710,194 and £179,526, respectively. Although challenging to modify in an emergency environment, information about wastage should be presented in multidisciplinary meetings between trauma and transfusion laboratory staff.

These data also provide a baseline from which to ascertain the cost-effectiveness of new interventions or transfusion protocols where the potential incremental cost implications can be complex and difficult to anticipate. For example, analysis of this study cohort (details available on request) showed almost a quarter of patients did not receive FFP alongside PRBCs which is now recommended in haemostatic resuscitation guidelines [11, 24]. Indeed a recently published trial advocated use of FFP, platelets, and PRBCs in a 1:1:1 ratio as a safe means of reducing death from exsanguination at 24 hours [8]. Whilst increasing adherence to such protocols is likely and can improve haemostasis rates, the impact on PRBC transfusion rates and overall patient survival is less clear. The data here for example, show that increasing or reducing PRBC transfusion by just one unit per patient for example could cost or save almost £1,000,000 (7,783 units at £123.31 per unit) at the national level. Furthermore, it is essential that additional costs that may manifest through any improved patient survival are also fully acknowledged. For example, patients in this cohort surviving the first 24 hours, incurred average healthcare costs that were £18,884 (95 % CI: £14,892 to £22,876, *P* <0.01) greater than patients who died within the first 24 hours (data not shown).

This study has limitations. There were missing data (Additional file 1: Table A1), but largely for low cost items, and established statistical techniques were used for imputation. Also, only care received by patients at study hospitals was considered, and so re-admissions to other hospitals were not captured. Sensitivity analysis was used to explore the potential impact of an underestimating of hospital re-admissions, and showed that even a three-fold increase in the rate observed increased total costs only slightly. Finally, the cost estimates presented here can only be considered an underestimate of the true costs to the NHS of treating severely bleeding trauma patients, as they do not include any day-case, outpatient, and primary care contacts during the 12 months following injury, which for this patient group are likely to be numerous. A recent study estimating comprehensive 12-month healthcare costs of a cohort of trauma patients reported that post-discharge costs accounted for 40 % of the total annual costs incurred [25]. If this finding were transferrable to the UK, total 12-month costs to the NHS of treating a major trauma patient with severe bleeding could be as high as £33,000.

## Conclusions

Severely bleeding major trauma patients are a small yet high cost subgroup of all major trauma patients, and costs are projected to rise further as a consequence of an aging population, and as evidence continues to emerge on the benefits of early and simultaneous administration of blood products in pre-specified ratios. The findings from this study provide a previously unreported baseline from which the potential impact of changes to service provision and/or treatment practice can begin to be evaluated. Further studies are required to determine the costs of post-discharge out-of-hospital care requirements, which are also likely to be substantial.

## Key messages

- This is the first study to use detailed resource use data from TARN, the independent monitor of trauma care in England and Wales, to estimate comprehensive treatment costs for severely bleeding trauma patients.
- The average cost of initial hospital treatment for a severely bleeding trauma patient is estimated to be £19,770 (SE: £810), of which almost 62 % is attributable to ventilation, ICU, and ward stays, and a further 16 % to surgery.
- The total cost to the NHS in England of acute treatment for the estimated 7,783 severely bleeding trauma patients presenting to hospital each year is calculated to be £148,293,657.
- Severely bleeding trauma patients are a small yet very high cost group of patients, and with an aging population, costs are projected to rise further.
- True costs will likely be higher than those reported here as the costs of primary and secondary care received post-hospital discharge were not included in this study. Further work is required to capture these costs in bleeding trauma survivors.

## Additional file

Additional file 1:
**This file provides supplementary information on the costing methods used in the study and on the techniques used to handle missing resource use data [** 17 **,** 18 **,** 29 **,** 30 **].** (DOCX 30 kb)

## Abbreviations

A&E: Accident and emergency
CI: Confidence interval
CLT: Central limit theorem
ED: Emergency department
EDCR: Electronic data collection and reporting
FFP: Fresh frozen plasma
HES: Hospital episode statistics
ICU: Intensive care unit
ISS: Injury severity score
IV: Intravenous
MHP: Massive haemorrhage protocol
MI: Multiple imputation
NHS: National health service
PRBCs: Packed red blood cells
SA: Sensitivity analysis
SE: Standard error
TARN: Trauma audit research network
