# Supplementary material for: Quantifying the healthcare costs of treating severely bleeding major trauma patients: a national study for England
Source: Crit Care. 2015 Jul 6;19(1):276. doi: 10.1186/s13054-015-0987-5 (PMC4517367; doi:10.1186/s13054-015-0987-5)
Supplement: Additional file 1: — This file provides supplementary information on the costing methods used in the study and on the techniques used to handle missing resource use data [ 17 , 18 , 29 , 30 ]. (DOCX 30 kb) [file 13054_2015_987_MOESM1_ESM.docx]

**Additional file**

**Supplementary information on costing methods**

*Trauma Team*

Medical and nursing personnel who comprise the trauma team were identified from the literature and verified using expert clinical opinion.[1] Team members included an A&E consultant (team leader), an anaesthetist, an anaesthetic assistant, a general surgeon, an orthopaedic surgeon, an A&E physician, two nurses, a radiographer, and a scribe (nurse). Staff salaries and working hours for trauma team members were taken from Curtis.[2] Salaries included on costs (employers’ superannuation and National Insurance contributions) and were divided through by working hours to give a cost per hour for each staff member, which was then used to cost the duration for which the trauma team attended each patient in the ED for up to a maximum of one hour (see main paper).

*Care received at other acute hospitals*

Six patients were transferred directly from the ED to another acute hospital (see Figure 1 main paper), and no further resource use data were collected. Of these six patients, three died following transfer (one after nine days, one within 30 days (date of death was unrecorded) and one after 41 days). These three patients were assumed to have been admitted to critical care and ventilated at the receiving hospital and to have remained there until death. For the three surviving patients, ICU, ward stay and ventilated days equivalent to the average observed for patients who spent their acute phase of care in the original treating hospital before being discharged home (or to a relative) or to a rehabilitation unit or a nursing home (n=242, see Figure 1 main paper) were assumed and costed.

Seventy patients were discharged to another acute hospital or institution after an inpatient stay at the original treating hospital. Of these 70 patients, five were known to have died; one after nine days, one after 11 days, one after 13 days, one after 16 days and one after 54 days. These patients were assumed to have been admitted to critical care and ventilated at the receiving hospital, and to have remained there until death. Fifty-four of the 70 patients discharged to another acute hospital or institution were alive at 12 months. The majority had received critical care and general ward care during their original hospital admission and so were assumed only to require ward care at the receiving hospital. Length of stay was assumed equivalent to the mean ward stay observed for patients who were discharged home (or to a relative), or to a rehabilitation unit or a nursing home from the original treating hospital (n=242, see Figure 1 main paper). The same assumption was made for the remaining 11 patients discharged to another acute hospital or institution, but for whom 12 month survival data were unavailable.

**Missing Data – Multiple Imputation**

Multiple imputation (MI) can be used to handle data which are missing at random (MAR), that is that the probability that data are missing is independent of unobserved values, conditional on the observed data.[3, 4]. MI utilises the distribution of the observed data to predict values of missing observations. This is done by using regression analyses to make such predictions but by also simultaneously acknowledging and accounting for uncertainty in the imputation process. Such ‘imputation’ uncertainty arises from two sources. Firstly there will be some variability between observations that is not explained by the regression model, but which is captured by the model’s error term. By ignoring such uncertainty, one would in effect be assuming that patients with the same values of coefficients would experience identical values of the dependent variable, which in reality is unlikely. Secondly, the model’s regression coefficients are themselves estimates and will therefore be subject to sampling variability. By incorporating this uncertainty into the imputation process one acknowledges that regression coefficients are not known quantities.

For this study a number of variables with missing data were present and individual regressions were specified for each of these variables. Regressions were tailored to the type of variable being predicted for example for continuous variables such as time in theatre linear regression (with prediction mean matching) was used whereas for categorical variables e.g. did the patient see a general surgery consultant, yes / no, a logistic regression was specified. Imputation was conducted in a chained or sequential manner which can be described in a number of steps:

1. Firstly and to be able to operationalise the approach, all missing data points were filled using simple sampling with replacement from the observed values.
2. For the first variable in the dataset with missing data, observed values were regressed on all other remaining variables (those with incomplete data, but also any variables with complete data). The resulting regression model had a set of coefficients β and a covariance matrix V which are assumed to follow a multivariate normal distribution.
3. A random draw from this distribution provided a set of coefficient values which were then used to predict the missing observations in the data for this variable (allowing the uncertainty in the imputation process to be addressed).
4. The programme then moved or switched to the next variable in the dataset with missing observations and regressed observed values of this variable on all other variables, including the previous variable and its imputed values.
5. A random draw from the resulting distribution of this model was then made and used to predict the missing observations in the data for this variable.
6. The process continued until all variables with missing data had been included (called a cycle).
7. The cycling process was then repeated 10 times in order to stabilise the results.
8. The whole process (steps 1 to 7) was repeated 20 times, thus generating 20 sets of imputed data.

The multiple imputation process was performed in Stata 12.1 (StataCorp, College Station, Texas) using the *mi impute chained* command.

**Table A1 Observed and imputed data for variables with some missing data**

|  | **Observed Data** | | **Imputed Data** | | |
| --- | --- | --- | --- | --- | --- |
|  | **n** | **%** | **Median number** | **Range** | **Overall percent** |
| **Transport to Hospital**  Own  Ambulance  Helicopter  Ambulance and helicopter  Unknown | 2  235  125  23  56 | 0.52  61.04  32.47  5.97  -- | 4  272  139  25  -- | 2-8  267-277  134-145  24-28  -- | 1.00  61.73  31.47  5.81  -- |
| **A&E Consultant contact**  Yes  No  Unknown | 353  87  1 | 80.23  19.77  -- | 354  87  -- | 353-354  87-88  -- | 80.25  19.75  -- |
| **Time in ED**  < 1hr  1 – 2 hrs  2 – 3 hrs  3 – 4 hrs  4 – 5 hrs  5 – 6 hrs  6+ hrs  Unknown | 92  67  82  56  48  20  40  36 | 22.72  16.54  20.25  13.83  11.85  4.94  9.88  -- | 99  73  91  61  53  21  43  -- | 94-103  71-76  85-95  58-65  49-61  20-26  42-46  -- | 22.26  16.55  20.51  13.87  12.00  4.98  9.84  -- |
| **Total time in theatre**  0 hrs  0 – 2 hrs  2 – 4 hrs  4 – 6 hrs  6 – 8 hrs  8 – 10 hrs  10 + hrs  Unknown* | 205  27  56  42  25  28  41  17 | 48.35  6.37  13.21  9.91  5.90  6.60  9.67  -- | 205  28  60  44  28  30  45  -- | --  27-30  58-62  43-47  25-30  29-32  43-47  -- | 46.49  6.35  13.56  10.08  6.36  6.96  10.20  -- |
| **Time in intensive care**  0 days  0 – 1 days  1 – 2 days  2 – 4 days  4 – 6 days  6 – 8 days  8 – 10 days  10 – 12 days  12 – 14 days  14 – 16 days  16 – 18 days   1. – 20 days   20 + days  Unknown | 91  58  36  50  34  34  17  14  13  13  12  10  49  10 | 21.11  13.46  8.35  11.60  7.89  7.89  3.94  3.25  3.02  3.02  2.78  2.32  11.37  - | 93  59  37  51  35  34  17  14  13  13  12  11  50  -- | 91-96  58-61  36-40  50-53  34-38  34-36  17-18  14-15  13-16  13-14  12-13  10-12  49-52  -- | 21.08  13.40  8.41  11.62  7.96  7.83  3.95  3.22  3.05  2.99  2.79  2.42  11.28  -- |
| **Time on wards**  0 days  0 – 1 days  1 – 5 days  5 – 10 days  10 – 15 days  15 – 20 days  20 – 25 days  25 – 30 days  30 – 35 days  35 – 40 days  40 – 45 days  45 – 50 days  50 + days  Unknown | 150  3  47  56  36  18  24  15  14  16  11  7  37  7 | 34.56  0.69  10.83  12.90  8.29  4.15  5.53  3.46  3.23  3.69  2.53  1.61  8.53  - | 155  3  47  56  36  18  24  15  14  16  11  7  37  - | 154-157  3-4  47-49  56-58  36-37  18-19  24-26  15-17  14-16  16-18  11-14  7-8  37-38  -- | 35.15  0.69  10.65  12.74  8.10  4.18  5.50  3.51  3.25  3.70  2.59  1.60  8.34  -- |
| **Time on ventilation**  0 days  0 – 1 days  1 – 2 days  2 – 3 days  3 – 4 days  4 – 5 days  5 – 6 days  6 – 7 days  7 – 8 days  8 – 9 days  9 – 10 days  10 + days  Unknown | 43  77  28  10  7  9  6  6  4  0  4  16  231 | 20.48  36.67  13.33  4.76  3.33  4.29  2.86  2.86  1.90  0  1.90  7.62  -- | 92  163  58  19  14  17  12  13  7  0  8  34  -- | 78-107  143-182  45-73  14-28  12-23  13-25  7-19  8-18  5-13  0  4-12  28-48  -- | 20.75  37.18  12.95  4.56  3.41  4.00  2.82  2.86  1.71  0.00  1.88  7.88  -- |
| **Full Blood Count**  0  1  2  3  4  5  6  Unknown | 4  102  30  31  15  5  1  253 | 2.13  54.26  15.96  16.49  7.98  2.66  0.53  -- | 9  249  76  71  29  8  1  -- | 5-19  229-263  60-91  53-81  22-33  5-19  1-3  -- | 2.11  56.25  17.10  15.51  6.56  2.14  0.33  -- |
| **Fibrinogen**  0  1  2  3  4  5  Unknown | 112  38  8  19  8  3  253 | 59.57  20.21  4.26  10.11  4.26  1.60  -- | 257  94  20  42  16  5  -- | 241-276  85-110  13-28  57-54  13-28  3-8  -- | 58.93  21.90  4.50  9.57  3.87  1.22  -- |
| **APTT**  0  1  2  3  4  5  6  Unknown | 97  36  16  24  10  4  1  253 | 51.60  19.15  8.51  12.77  5.32  2.13  0.53  -- | 225  92  39  55  22  6  1  -- | 208-246  81-104  29-51  44-65  14-29  4-13  1-5  -- | 51.13  20.74  8.95  12.47  4.88  1.45  0.39  -- |
| **Prothrombin**  0  1  2  3  4  5  6  Unknown | 60  61  22  23  16  5  1  253 | 31.91  32.45  11.70  12.23  8.51  2.66  0.53  -- | 149  144  57  43  32  9  1  -- | 131-165  127-176  43-75  35-55  26-38  6-19  1-3  -- | 33.76  32.77  13.30  10.37  7.30  2.17  0.36  -- |
| **INR**  0  1  2  3  4  5  Unknown | 131  31  12  7  5  2  253 | 69.6816.496.38  3.72  2.66  1.06  -- | 315  73  25  17  8  3  -- | 291-325  60-83  18-39  10-23  5-13  2-6  -- | 71.12  16.55  6.04  3.61  1.96  0.71  -- |
| **APTTr**  0  1  2  3  4  5  Unknown | 154  17  7  5  4  1  253 | 81.919.04  3.72  2.66  2.13  0.53  -- | 375  35  13  9  7  1  -- | 354-381  29-47  8-23  6-18  4-14  1-3  -- | 84.54  8.16  3.17  2.18  1.64  0.31  -- |
| **CT Scan**  0  1-2  3-4  5-6  7-8  9-10  10+  Unknown | 111  245  68  12  2  1  1  1 | 25.23  55.68  15.46  2.73  0.45  0.23  0.23  -- | 111  245  68  12  2  1  1  -- | 111-112  245-246  68-69  12-13  --  --  --  -- | 25.18  55.61  15.52  2.78  0.45  0.23  0.23  -- |
| **X-ray**  0  1-2  3-4  5-6  7-8  9-10  10+  Unknown | 90  273  48  12  6  3  8  1 | 20.4562.0510.912.73  1.36  0.70  1.82  -- | 90  273  48  12  6  3  8  -- | 90-91  273-274  48-49  12-13  6-7  --  8-9  -- | 20.44  61.98  10.93  2.73  1.37  0.68  1.86  -- |
| **Ultrasound**  0  1  2  Unknown | 408  29  3  1 | 92.73  6.59  0.68  -- | 409  29  3  -- | 408-409  29-30  --  -- | 92.72  6.60  0.68  -- |
| **MRI**  0  1  2  3  Unknown | 416  21  2  1  1 | 94.55  4.77  0.45  0.23  -- | 417  21  2  1  -- | 416-417  21-22  2-3  --  -- | 94.52  4.78  0.46  0.23  -- |
| **PRBC units wasted**  0  1 - 3  4 – 9  10 +  Unknown | 254  35  9  3  140 | 84.39  11.63  2.99  0.99  -- | 361  54  18  6  -- | 356-373  45-60  12-24  3-14  -- | 82.38  11.94  4.22  1.46  -- |
| **FFP units wasted**  0  1 - 3  4 – 9  10 +  Unknown | 239  42  31  1  128 | 76.36  13.42  9.90  0.32  -- | 331  59  49  2  -- | 307-338  54-67  42-66  1-5  -- | 74.65  13.56  11.30  0.49  -- |
| **Platelet units wasted**  0  1 - 3  4 – 9  10 +  Unknown | 283  16  0  0  142 | 94.65  5.35  0  0  -- | 404  36  0  0  -- | 393-413  28-48  0  0  -- | 91.75  8.25  0  0  -- |
| **Cryoprecipitate units wasted**  0  1 - 3  4 – 9  10 +  Unknown | 260  34  5  0  142 | 86.96  11.37  1.67  0  -- | 374  54  9  0  -- | 363-385  48-68  5-18  0  -- | 85.06  12.76  2.19  0  -- |
| **Dextrose (mls) in first 24 hours**  0  1 - 500  500 – 1000  1000 – 2000  2000 – 3000  Unknown* | 425  1  5  4  0  6 | 97.70  0.22  1.15  0.92  0  -- | 425  1  7  7  0  -- | --  1-4  5-11  4-10  --  -- | 96.37  0.42  1.59  1.62  0  -- |
| **Colloids (mls) in first 24 hours**  0  1 - 500  500 – 1000  1000 – 2000  2000 – 3000  3000 – 4000  4000 – 5000  5000 – 6000  6000 +  Unknown* | 303  27  33  24  12  8  1  4  3  26 | 73.01  6.51  7.95  5.78  2.89  1.93  0.24  0.96  0.72  -- | 303  32  41  30  15  10  1  5  4  -- | --  29-35  37-45  25-34  13-17  8-12  1-2  4-8  3-6  -- | 68.71  7.15  9.29  6.85  3.41  2.20  0.29  1.11  0.99  -- |
| **Crystalloids (mls) in first 24 hours**  0  1 - 500  500 – 1000  1000 – 2000  2000 – 3000  3000 – 4000  4000 – 5000  5000 – 6000  6000 – 7000  7000 +  Unknown* | 170  30  39  56  43  36  15  8  6  5  33 | 41.67  7.35  9.56  13.73  10.54  8.82  3.68  1.96  1.47  1.23  -- | 170  34  42  63  48  42  17  9  7  6  -- | --  30-37  39-46  59-69  44-53  38-47  15-21  8-16  6-10  5-10  -- | 38.55  7.73  9.72  14.26  10.90  9.60  3.90  2.29  1.62  1.43  -- |
| **Starch (mls) in first 24 hours**  0  1 - 500  500 – 1000  Unknown* | 436  3  1  1 | 99.09  0.68  0.23  -- | 436  4  1  -- | --  3-4  1-2  -- | 98.87  0.83  0.31  -- |
| **Hypertonic saline (mls) in first 24 hours**  0  1 - 500  500 – 1000  1000 – 2000  2000 – 3000  3000 – 4000  4000 – 5000  5000 +  Unknown* | 354  15  17  21  8  5  4  3  14 | 82.90  3.51  3.98  4.92  1.87  1.17  0.94  0.70  -- | 354  17  20  25  9  6  5  3  -- | --  15-21  18-24  22-32  8-12  5-8  4-6  3-5  -- | 80.27  3.83  4.73  5.76  2.12  1.39  1.12  0.77  -- |
| **Hartmanns (mls) in first 24 hours**  0  1 - 500  500 – 1000  1000 – 2000  2000 – 3000  3000 – 4000  4000 – 5000  5000 – 6000  6000 +  Unknown* | 255  8  50  33  19  17  12  4  14  29 | 61.89  1.94  12.14  8.01  4.61  4.13  2.91  0.97  3.40  -- | 255  9  59  38  23  19  14  5  17  -- | --  8-12  55-65  34-44  20-26  17-22  13-17  4-8  14-19  -- | 57.82  2.13  13.47  8.75  5.25  4.42  3.28  1.10  3.78  -- |
| **Neurology Consultant contact**  Yes  No  Unknown | 17  423  1 | 3.86  96.14  -- | 17  424  -- | 17-18  423-424  -- | 3.90  96.10  -- |
| **General Surgery Consultant contact**  Yes  No  Unknown | 42  398  1 | 9.55  90.45  -- | 42  399  -- | 42-43  398-399  -- | 9.56  90.44  -- |
| **Cardiology Consultant contact**  Yes  No  Unknown | 12  428  1 | 2.73  97.27  -- | 12  429  -- | 12-13  428-429  -- | 2.77  97.23  -- |
| **Hospital re-admissions within 12 months** **– denominator n=316 survivors to hospital discharge**  Yes  No  Unknown | 25  256  35 | 8.90  91.10  -- | 26  290  -- | 25-29  287-291  -- | 8.35  91.65  -- |

*Patients known to have received treatment but duration / volume not recorded

**References**

1. Trauma.org. Resuscitation: The Trauma Team. http://www.trauma.org/archive/resus/traumateam.html. Accessed 12/10/2013.

2. Curtis L. Unit Costs of Health and Social Care 2013. Canterbury: Personal and Social Services Research Unit, University of Kent;2013.

3. White IR, Royston P, Wood AM. Multiple imputation using chained equations: Issues and guidance for practice. Stat Med. 2011;30(4):377-99.

4. Little R, Rubin D. Statistical Analysis with Missing Data. New York: J. Wiley and Sons; 1987.
